# BBP: *Brucella *genome annotation with literature mining and curation

**DOI:** 10.1186/1471-2105-7-347

**Published:** 2006-07-16

**Authors:** Zuoshuang Xiang, Wenjie Zheng, Yongqun He

**Affiliations:** 1 Unit for Laboratory Animal Medicine, University of Michigan Medical School, Ann Arbor, MI 48109, USA; 2 Webmail.us Inc., Blacksburg, VA 24060, USA; 3 Department of Microbiology and Immunology, University of Michigan Medical School, Ann Arbor, MI 48109, USA; 4 Bioinformatics Program, University of Michigan Medical School, Ann Arbor, MI 48109, USA

## Abstract

**Background:**

*Brucella *species are Gram-negative, facultative intracellular bacteria that cause brucellosis in humans and animals. Sequences of four *Brucella *genomes have been published, and various *Brucella *gene and genome data and analysis resources exist. A web gateway to integrate these resources will greatly facilitate *Brucella *research. *Brucella *genome data in current databases is largely derived from computational analysis without experimental validation typically found in peer-reviewed publications. It is partially due to the lack of a literature mining and curation system able to efficiently incorporate the large amount of literature data into genome annotation. It is further hypothesized that literature-based *Brucella *gene annotation would increase understanding of complicated *Brucella *pathogenesis mechanisms.

**Results:**

The *Brucella *Bioinformatics Portal (BBP) is developed to integrate existing *Brucella *genome data and analysis tools with literature mining and curation. The BBP InterBru database and *Brucella *Genome Browser allow users to search and analyze genes of 4 currently available *Brucella *genomes and link to more than 20 existing databases and analysis programs. *Brucella *literature publications in PubMed are extracted and can be searched by a TextPresso-powered natural language processing method, a MeSH browser, a keywords search, and an automatic literature update service. To efficiently annotate *Brucella *genes using the large amount of literature publications, a literature mining and curation system coined Limix is developed to integrate computational literature mining methods with a PubSearch-powered manual curation and management system. The Limix system is used to quickly find and confirm 107 *Brucella *gene mutations including 75 genes shown to be essential for *Brucella *virulence. The 75 genes are further clustered using COG. In addition, 62 *Brucella *genetic interactions are extracted from literature publications. These results make possible more comprehensive investigation of *Brucella *pathogenesis. Other BBP features include publication email alert service, *Brucella *researchers' contact database, and discussion forum.

**Conclusion:**

BBP is a gateway for *Brucella *researchers to search, analyze, and curate *Brucella *genome data originated from public databases and literature. *Brucella *gene mutations and genetic interactions are annotated using Limix leading to better understanding of *Brucella *pathogenesis.

## Background

*Brucella *is a Gram-negative, facultative intracellular coccobacillus which causes brucellosis in humans and animals [1]. *Brucella *are taxonomically placed in the alpha-2 subdivision of the class Proteobacteria. Traditionally there are six species of *Brucella *based on the preferential host specificity: *B. melitensis *(goats), *B. abortus *(cattle), *B. suis *(swine), *B. canis *(dogs), *B. ovis *(sheep) and *B. neotomae *(desert mice); two new species *B. cetaceae *(cetacean) and *B. pinnipediae *(seal) have recently been discovered [2]. The first four species are pathogenic to humans in decreasing order of severity making brucellosis a zoonotic disease. These *Brucella *species have been identified as priority agents amenable for use in biological warfare and bio-terrorism and listed as CDC/NIAID category B priority pathogens.

Complete genome sequences of 4 *Brucella *strains are currently available [3-6]. A typical *Brucella *genome usually has two circular chromosomes of approximately 2.1 MB and 1.2 MB. There are approximately 3,200 – 3,400 genes in each genome. The DNA sequences of different *Brucella *spp. share greater than 90% identity [4,6,7]. Genome sequences and annotated data are publicly available from existing databases such as RefSeq [8], Swissprot [9], and the TIGR Comprehensive Microbial Resource (CMR) [10]. These databases come from different sources and have different focuses. Different data visualization and analysis tools are also available in these database systems and other genome analysis systems. A web portal that integrates these data and analysis resources will greatly help *Brucella *gene research.

*Brucella *genome data in current databases is largely derived from computational analysis without literature support. It is partially due to the lack of a literature mining and curation system. The large amount of literature data can be used to not only validate the data obtained from computational analysis but also provide new insights not available from computational analysis. Literature mining techniques are being developed rapidly in the context of the genomic fields [11,12]. For example, Hu et al., [13] describe a rule-based system, RLIMS-P, for literature mining and database annotation of protein phosphorylation from MEDLINE abstracts. Stephens et al., [14] present an association and function discovery method to extract gene-gene interactions from co-occurring genes in MEDLINE abstracts. Hoffmann et al. [12] list more than 20 main text mining repositories and systems that are currently available. Compared to basic keyword search, many effective literature retrieval programs connect textual evidence to ontologies as main repository of formally represented knowledge. Ontologies are conceptual models that support consistent and unambiguous knowledge sharing and provide a framework for knowledge integration. TextPresso is a natural language processing (NLP) and ontology-based literature search engine with significant efficiency in biomedical literature retrieval [15]. Since computational literature mining techniques (e.g., TextPresso) still cannot guarantee precise retrieval, time consuming manual literature curation is required to obtain accurate results for database storage. It is possible for manual curation and computational text mining to work together for rapid retrieval and analysis of facts with standardization of the extracted information [16]. The PubSearch literature curation software is a literature curation management system with a powerful manual curation capability [17]. Our strategy of integrating different computational text mining tools including a TextPresso-powered program with a PubSearch-powered manual curation system has led to the development of a literature mining and curation system coined "Limix" that is currently applied to *Brucella *genome annotation.

The brucellae infect phagocytic macrophages and nonphagocytic epithelial cells (e.g., HeLa cells) *in vivo *and *in vitro *[18-20]. *Brucella *virulence relies on its ability of intracellular survival and replication. It is still unclear how many *Brucella *genes are essential for intracellular virulence and how virulent *Brucella *genes interact. It is hypothesized that mechanisms of *Brucella *pathogenesis can be better understood by systematically annotating *Brucella *gene mutations and genetic networks from all *Brucella *literature papers.

We have developed the *Brucella *Bioinformatics Portal (BBP) with focus on integrating *Brucella *genome data and analysis tools from existing resources and annotating *Brucella *genes and gene-gene interactions from literature publications. The updated information allows more comprehensive examination of *Brucella *pathogenesis. These genome annotation systems, together with other programs including publication email alert, *Brucella *researchers' contact database, and discussion forum, makes BBP an ideal bioinformatics portal for the *Brucella *research community. The BBP website is publicly available [see Additional file 1].

## Results and discussion

### System architecture

**Figure 1:**
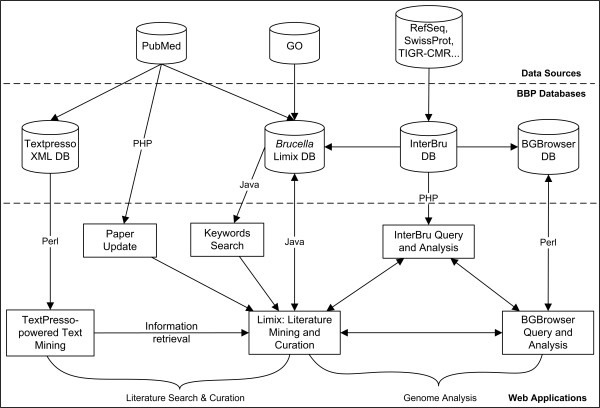
**The BBP system architecture for *Brucella *genome analysis and literature mining and curation**. A PubMed literature extraction and parsing program loads all *Brucella*-related papers from PubMed into the *Brucella *Limix database and the TextPresso-powered text processing pipeline. An automatic literature update program also extracts *Brucella *papers published in the recent and previous months. The Limix system provides an efficient way for literature searching and data extraction, edition, and submission by integrating computational text mining programs with manual literature curation and management features. InterBru integrates *Brucella *genome data from different data sources including our in-house curated data from the *Brucella *Limix database. The *Brucella *Genome Browser (BGBrowser) features graphic visualization of *Brucella *genome data and offers many analysis tools. InterBru and BGBrowser also share the same output page displaying comprehensive *Brucella *gene and protein information.

A three-tier system architecture is implemented with two Linux servers (Figure 1). Users submit database or analysis queries using front-end web browsers via HTML forms. These requests are processed using PHP/Java/Perl/(middle-tier, application server) against the Oracle relational database (back-end, database server), or XML and MySQL databases in application server. The result of each query is then presented to the users through the web browser. The BBP Oracle database stores all the data schema and data for the programs developed in-house, including the literature MeSH data, ContactsDB, registration information, and Forum data. The *Brucella *Limix and BGBrowser databases are implemented in the application server using MySQL since both systems are modified from open sources with MySQL as the default database management system. The TextPresso XML database is also implemented in the application server. Table 1 shows all the data and analysis resources incorporated by BBP.

### *Brucella *genome data query, browsing, and analysis

**Figure 2:**
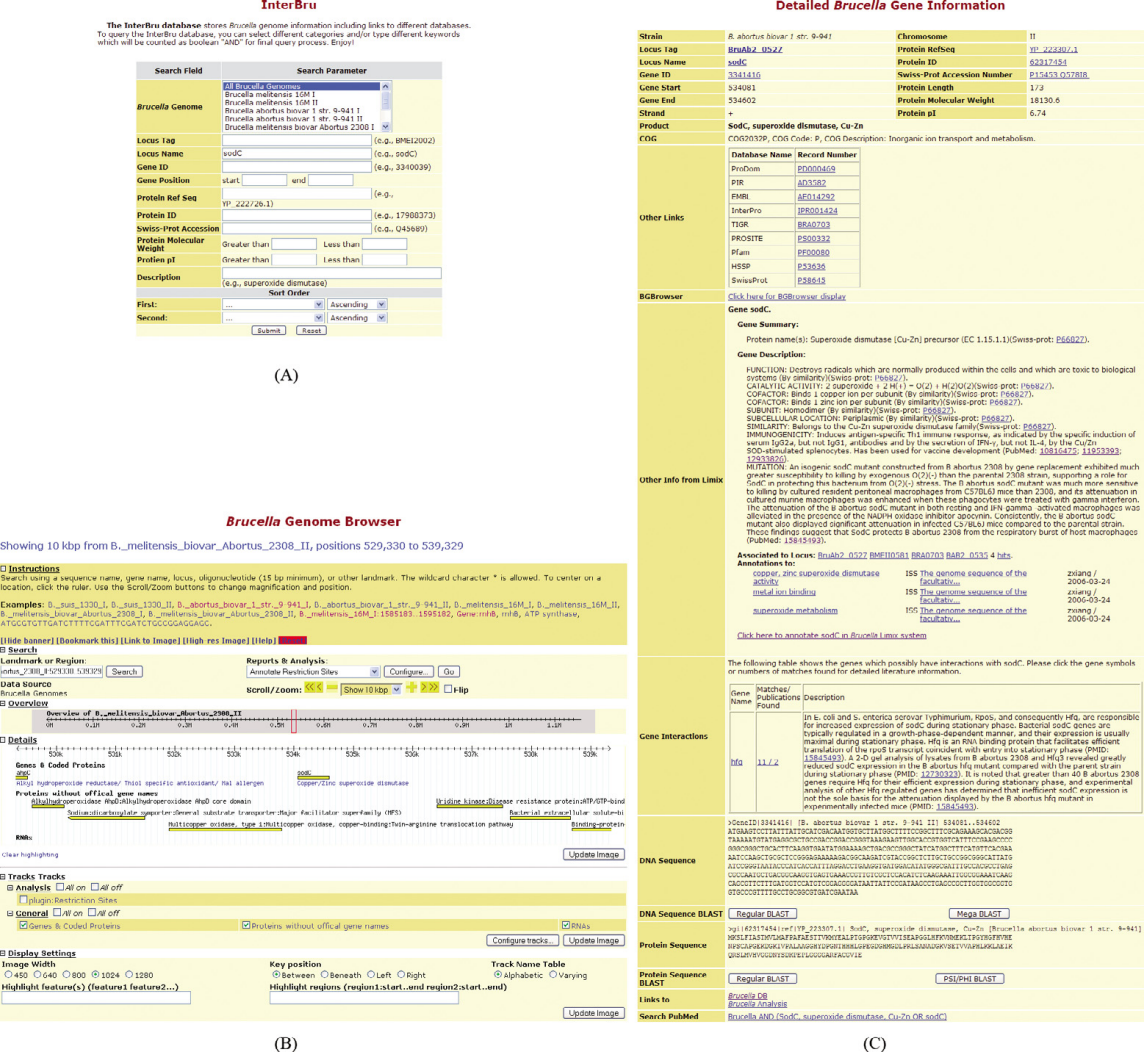
**A scenario of *Brucella *genome query and analysis**. (A) The InterBru database allows users to search public databases (e.g., RefSeq, Swissprot) for *Brucella *genes and proteins via different characteristics or identifiers. Here a user searches for *Brucella sodC *gene. (B) BGBrowser localizes the *sodC *gene and it neighbor genes in *Brucella *genomes and provides many add-on gene analysis tools. (C) The detailed gene information table shared by InterBru and BGBrowser provides sequences and functional annotation of *Brucella sodC *gene and its encoded protein Cu/Zn superoxide dismutase. Links to various databases and detailed curated data from Limix are summarized. Local BLAST programs are also available from this page for similarity analysis.

Two complementary programs, the InterBru database system and *Brucella *genome browser (BGBrowser), have been developed for *Brucella *genome data query, browsing, and analysis. Both programs allow query of *Brucella *gene data from all four complete genomes: *B. melitensis *16 M [5], *B. suis *1330[3], and *B. abortus *strain 9–941 [4] and strain 2308 [6]. The InterBru web query interface allows users to search *Brucella *genes based on different gene features such as gene name, locus tag, protein molecular weight (MW) and isoelectric points (PI), RefSeq identifier, and Swissprot accession number (Figure 2A). The Generic Genome Browser, also known as GBrowse [21], is a popular genome browser tool due to its portability, simple installation, and convenient data input and easy integration with other software programs. Developed as a member of the GBrowse family, the BBP BGBrowser program provides web query interface and graphic representation of specific *Brucella *genes, proteins, and RNA features (Figure 2B). BGBrowser also provides many data analysis programs for tasks such as annotating restriction sites, finding short oligos, and downloading protein or DNA sequence files. Both InterBru and BGBrowser share the same gene information page, which contains detailed *Brucella *gene and protein information and links to many databases and analysis programs (Figure 2C).

The following is a typical scenario when a *Brucella *researcher searches for more information about *B. abortus sodC *gene encoding Cu/Zn superoxide dismutase (SOD). The user starts with querying "*sodC*" gene in InterBru (Figure 2A). Four *Brucella sodC *genes from 4 *Brucella *genomes will be found, including one from *B. abortus *strain 2308 and one from *B. abortus *strain 9–941. The detail information about the *sodC *gene in strain 2308 is shown in the detailed *Brucella *gene information page (Figure 2C). This page includes basic gene information and through unique database identifiers links to many public databases, such as RefSeq, GenBank, Swissprot, InterPro, and PubMed. This page also contains *sodC*-specific gene annotation and genetic interaction data curated by the BBP team from literature using the *Brucella *Limix system. A link to the *Brucella *Limix is also available for users to annotate *sodC *gene. A direct link to PubMed allows users to access all *Brucella sodC*-related publications. Both DNA and protein sequences are provided with additional links to internal BLAST search services (regular Blast, Psi/Phi Blast, and Mega Blast) where different *Brucella *nucleotide and protein sequence libraries have been created for convenient use. For example, a simple Blastn search indicates that the *sodC *DNA sequence in *B. abortus *strain 2308 is 100% identical to that in *B. abortus *strain 9–941 but 99% identical to that in *B. melitensis *strain 16 M and *B. suis *strain 1330. The protein sequences in the four genomes are 100% identical to each other. The user is also directed to the BGBrowser to inspect the genes next to *sodC *in the genome, annotate restriction sites, or perform other analyses (Figure 2B). To get more information, the user can submit questions in the BBP discussion Forum or email to the *Brucella *listserv.

### *Brucella *literature search

Four computational literature search methods have been developed to search *Brucella *literature: TextPresso for *Brucella*, MeSH browser, keyword search, and automatic *Brucella *publication update.

Textpresso is an information retrieval system available from the Generic Software Components for Model Organism Databases (GMOD) [22]. It splits papers into sentences and further to XML-tagged words or phrases, which are classified using categories of ontology. The specifically designed ontology can be used to query information on specific classes of biological concepts (e.g., gene, mutant) and their relationships (e.g., association, regulation). It has been used in WormBase [23] and many other projects [24]. We have adopted and extended TextPresso for *Brucella *literature text mining. Currently it stores abstract information of 3930 *Brucella *publications. Among them 1083 papers have full-text contents. While it takes approximately 24 hours for TextPresso to preprocess these 3930 PubMed abstracts and 1083 full text PDF files in our server, the online query process is fast (~0.5 sec/query).

**Figure 3:**
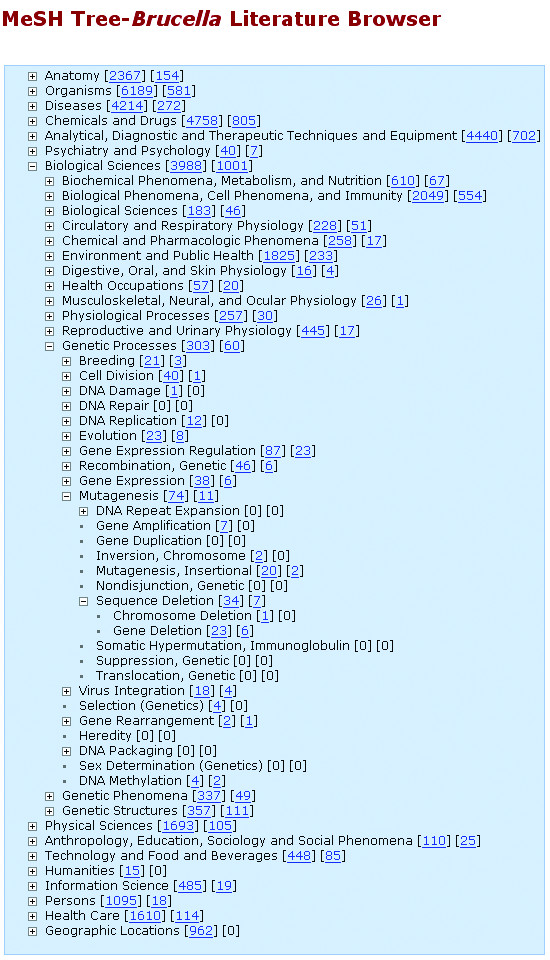
**MeSH Browser**. All the *Brucella *literature publications can be visualized by the interactive MeSH-tree browser. The two clickable numbers in each line links to all publications with the term as a MeSH term or a major MeSH term, respectively. This figure shows the hierarchical MeSH tree structure leading to Mutagenesis and Gene Deletion.

MeSH is the controlled vocabulary of medical and scientific terms assigned by experts and used for indexing articles in PubMed. MeSH terminology provides a consistent approach to retrieve information that may use different terminology for the same concepts. The BBP MeSH browser enables users to locate *Brucella *articles by the MeSH terms in the hierarchical MeSH tree structure. Figure 3 illustrates the detailed tree display for those who want to search for gene deletion.

A user can also search the locally built *Brucella *literature database by keywords such as author, journal, year, issue, and abstract. Although the *Brucella *literature database is updated periodically, it may miss the newest *Brucella *literature publications. In order to capture this portion of the literature, a BBP internal program has been developed to automatically extract the newly published *Brucella *papers from PubMed.

### *Brucella *literature mining and curation system (Limix)

**Figure 4:**
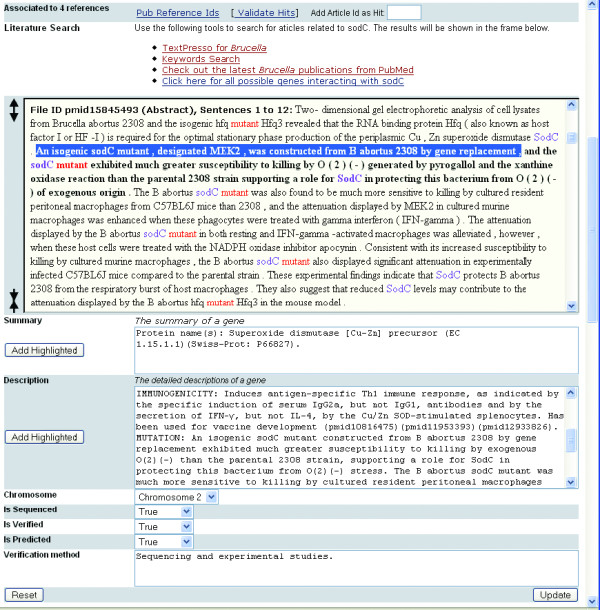
**Integrated computational text mining and manual curation in Limix**. The computational text mining frame shows a typical TextPresso-type result after query for the *sodC *keyword and "mutant" category. All *sodC *words and words under mutant category are clearly labeled in colors. One sentence containing both *sodC *and mutant words is highlighted in bold and considered as one match. A curator can easily highlight and copy text from this frame to an editable text field below the frame within the same page. The data can be further edited and submitted to a backend database by clicking an 'update' button. Other literature retrieval approaches (e.g., keywords search) are also available in the computational text mining frame.

Although the text mining approaches efficiently provide queried articles and even sentences, the retrieved results are not precise and cannot be directly edited and stored in database. By contrast, a manual literature curation and management system usually allows edited literature data to be stored in database. The *Brucella *Limix system is developed through integrating literature text mining technologies (including TextPresso for *Brucella*, keywords search, and latest literature updates) and the PubSearch-powered manual literature curation and management program. Within one web page, a data curator is able to perform computational text mining, copy highlighted text from the computational search to an editable text field, edit, and further submit reviewed results to the backend database (Figure 4). Limix allows curators to conveniently search, update, validate and insert gene information. Figure 4 shows an example of using Limix to search and annotate phenotypes of a *sodC *mutation from *Brucella *literature. Limix is also a distributed curation system that is capable of involving external experts to support our curation efforts. Direct submissions from scientists will help keep the database as comprehensive, updated and accurate as possible.

### Literature-curated *Brucella *gene mutations and pathogenesis

We have applied the *Brucella *Limix system for annotation of more than 900 *Brucella *genes. Out of more than 200 possible gene mutations from TextPresso-powered computational search, 107 mutations are manually confirmed, and 75 mutated genes are found to be attenuated inside macrophages or HeLa cells, or in an *in vivo *mouse model. It suggests that these 75 mutated *Brucella *genes are essential for *Brucella *virulence and pathogenesis. Although this list does not include those genes with attenuated mutation phenotype but without defined gene names, the number of attenuated mutations we have found is much more than any single research or review paper has discussed. The NCBI Clusters of Orthologous Groups (COGs) approach provides phylogenetic classification of proteins encoded in complete genomes [25]. The 75 *Brucella *genes are classified using the COG method for further analysis (Table 2). It first confirms the well-known pathogenesis mechanisms of *Brucella *type IV secretion system encoded by the virB operon [26], the BvrR-BvrS two-component regulatory system encoded by *bvrR *and *bvrS *[27], and the complete *Brucella *lipopolysaccharide [28]. Significant and stable attenuation are obtained in *Brucella *strains with mutations (e.g., *wboA*) resulting in the loss of normal lipopolysaccharide O-side-chain biosynthesis [29]. In addition, our curation clearly indicates the critical importance of transport and metabolism of various metabolites including amino acid, carbohydrate, lipid and inorganic ions (Table 2). Since the brucellae survive inside phagosomes of eukaryotic cells, bacterial attenuation after disruption of these genes suggests that the corresponding metabolites are not accessible to the bacteria inside the phagosomes, but they are essential for intracellular growth. Limix has also uncovered many gene mutations with important implications in understanding *Brucella *pathogenesis. For example, studies with a *B. abortus sodC *mutant suggest that Cu/Zn SOD protects *B. abortus *from respiratory burst of host macrophages [30]. The presence of an attenuated *fliF *mutant suggests a possible role for flagella in virulence [31], and it further leads to the recent discovery of a polar and sheathed flagellar structure in the early log phase of a growth curve in 2YT nutrient broth [32]. This finding has changed previous dogma that non-motile *Brucella *species do not have functional flagella.

### Literature-curated *Brucella *genetic interactions and pathogenesis

*Brucella *pathogenesis relies on interactions between individual *Brucella *genes. Besides individual *Brucella *gene mutations, we have also analyzed *Brucella *genetic interactions using all accessible *Brucella *literature publications. As defined in the original TextPresso paper [15], *Brucella *genetic interactions are retrieved using a TextPresso-powered method to search for sentences containing >= 2 'gene', and >= 1 'association' or >= 1 'regulation' categories. Such a sentence is counted as one match. A program is developed to run pairwise searching of *Brucella*-related publications for every two *Brucella *genes from 951 *Brucella *genes obtained from NCBI and EBI databases. Manual curation is performed to confirm if a possible interaction hit is true (i.e., a true positive) and to assign a gene ontology (GO) evidence code indicating the evidence of the finding [17]. Table 3 indicates that the number of true genetic interactions found in Limix depends on how many matches and publications are counted as the cutoffs for TextPresso search and if full text contents are searched for in addition to abstracts. On the condition that only one match is required for positive hits during computational text mining, 58 out of 1330 possible genetic interactions (true positive rate is 4.4% (58/1330)) are confirmed to be true interactions if both abstracts and full text contents are used, and only 17 out of 38 genetic interactions are confirmed to be true (true positive rate is 44.7% (17/38)) if only abstracts are considered (Table 3). This indicates that inclusion of full text contents results in more confirmed results (58 vs. 17), while inclusion of only abstracts leads to higher true positive rate (44.7% vs. 4.4%). It is possible to significantly increase true positive rate by raising the searching threshold of the number of matches in case both abstracts and full text contents are used. For example, the true positive rate becomes 23.5% (50/213) if the cutoff becomes 2 matches from at least one paper (Table 3).

Limix also allows curators to add *Brucella *genetic interactions that are not detected by the TextPresso-based text mining approach. Currently 62 genetic interactions are available in the Limix databases. There are 48 genes involved in these interactions, and 28 of them are shared with the attenuated *Brucella *gene mutation list as discussed above. The finding of these genetic interactions has provided more comprehensive investigation of *Brucella *pathogenesis. For example, it not only confirms the importance of type IV secretion system and the BvrR-BvrS two-component regulatory system in *Brucella *pathogenesis but also provides specific pathway details. Furthermore, our curation results indicate that the secretion of the N-terminal fragment of BvrR fused to a CAT report gene is diminished in *virB1 *and *virB10 *mutants, suggesting that BvrR is probably an effector protein secreted by the VirB type IV secretion system [33]. Another interesting observation is the interactions among *sodC*, *hfq*, and *ctrA*. *B. abortus *host factor 1 (HF-1) protein encoded by *hfq *contributes to stress resistance during stationary phase and is a major determinant of virulence in mice [34]. Bacterial *sodC *genes are typically regulated in a growth-phase-dependent manner, and their expression is usually maximal during stationary phase. *B. abortus hfq *gene mutation results in greatly reduced *sodC *expression [35]. CtrA is a master response regulator that is essential for viability and is transcriptionally autoregulated. The *hfq *gene is likely to be negatively regulated by CtrA [36]. These two interactions suggest that CtrA may also regulate *Brucella sodC *expression.

**Figure 5:**
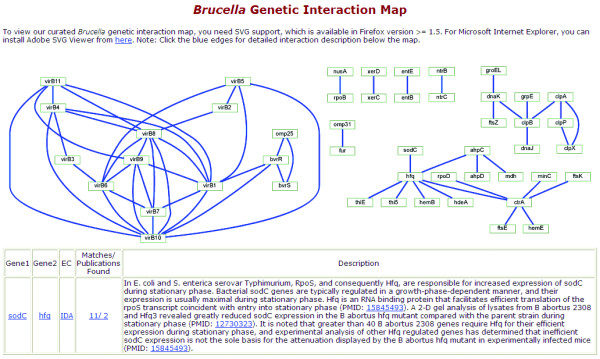
***Brucella *genetic interaction map and description**. Limix is used to find and confirm 62 *Brucella *genetic interactions. In the *Brucella *genetic interaction map displayed in a SVG form, any node can be clicked for detailed gene information, and any edge can be clicked to show description of the specific interaction.

A software program based on Graphviz [37] is developed to display all the genetic interactions in the Scalable Vector Graphics (SVG) format [38] (Figure 5). SVG is a language for describing two-dimensional graphics and graphical applications in XML and is currently supported by many internet browsers. A click on each node in the map will link to the detailed gene information page in InterBru search. Once an edge (straight line) is clicked, the detail on the specific gene-gene interaction is shown. Figure 4 demonstrates the interaction between two *Brucella *genes *sodC *and *hfq*. A future direction is to integrate our curated genetic interaction data with known interaction and pathway knowledge from existing databases, such as KEGG [39], BIND [40], and DIP [41].

### Other portal features: ContactsDB, Forum, and publication Email alert service

BBP is designed to link international *Brucella *scientists and researchers. BBP contains a ContactsDB database that currently provides contact information for more than 100 *Brucella *researchers in the world. The ContactsDB can be queried based on first name, last name, address, city, institute, state, zip code, and country. Any *Brucella *researcher can also enter new contact information or update existing information using an interactive web page. The BBP discussion Forum has been created to facilitate discussion between scientists. Only registered BBP members can initiate a topic, reply to a message, or edit their own messages. Unregistered users can view all discussions. Up to now more than 50 *Brucella *researchers from 18 different countries have registered in BBP. Another BBP feature is the Publication Email Alert Service. This service automatically notifies users of newly published papers within a user-defined time interval. Those users who have not registered for this service can view new publications by visiting our automatic new *Brucella *paper updating website.

## Conclusion

Many different databases related to *Brucella *genomes and genes exist. A variety of computational tools are also available for functional genomic analysis. The *Brucella *Bioinformatics Portal is a gateway to provide or link functional *Brucella *gene information and analysis tools useful for the *Brucella *researchers. Besides summarizing *Brucella *genomics related databases and analysis tools in HTML formats, we have also developed the InterBru database and the *Brucella *Genome Browser (BGBrowser). InterBru allows users to search for specific *Brucella *gene information and provide links to existing databases. BGBrower provides graphic visualization and analysis tools. Since most of current *Brucella *genes and gene-gene interaction data are derived from computational analysis and often lack literature support, we further developed several computational *Brucella *literature search tools for efficient retrieval of *Brucella *articles. The *Brucella *Limix system is also developed to allow retrieved data from text mining tools to be directly copied, edited and submitted to a backend relational database. The *Brucella *Limix system has been used to annotate a large number of *Brucella *genes and to find 62 *Brucella *genetic interactions and 75 attenuated gene mutations from literature publications in PubMed. These annotated results provide more comprehensive understanding of *Brucella *pathogenesis. These programs, together with other portal features including the ContactsDB and Forum, facilitate the *Brucella *research community to obtain and annotate *Brucella *genome sequences in one website. BBP is the first integrated system for *Brucella *genome analysis.

BBP adopts and extends many open-source software programs for *Brucella *genome annotation including three GMOD open-source software programs, GBrowse, TextPresso, and PubSearch (Table 1). Many interactive graphical interfaces (e.g., MeSH browser and genetic interaction map) have also been developed for efficient literature mining and database curation. While many NLP-based text mining tools (e.g., TextPresso) significantly improve the capability of biomedical text mining, an automatic literature retrieval tool that can be as accurate as manual literature curation still does not exist [12]. As far as we know, among existing web-based dedicated genome databases, BBP is the first to strongly integrate a literature manual curation and management system (e.g., PubSearch) with NLP-based computational literature mining techniques (e.g., TextPresso for *Brucella*) into an efficient literature mining and curation system (Limix). The BBP Limix system also provides a genetic solution for annotating other genomes and genes based on published literature data.

## Methods

### Server and programming tools

This BBP system is built on two Dell Poweredge 2580 servers, one serving as database server and another as application server. Both servers are running the Redhat Linux operating system (Redhat Enterprise Linux ES 4). The database server is powered by Oracle 10g database management system. Two open source software programs, Apache HTTP Server and Apache Tomcat, are installed as the HTTP application server and the servlet container respectively. Different programming languages including PHP, Perl, and Java are implemented for development of a variety of BBP modules. The two servers also back each other regularly to secure the data.

### InterBru and BGBrowser

InterBru is a web-based relational database system that contains various *Brucella *data and links to public databases. The protein MW and PI are calculated from the protein sequences using the modules (Bio::Tools::pICalculator and Bio::Tools::SeqStats) from Bioperl [42]. The InterBru data can be searched by different features and sorted for proper display (Figure 2A). The *Brucella *Genome Browser (BGBrowser) (Figure 2B) is developed based on the GBrowse [21], one of the GMOD software programs [22]. In order to speed up the query process, all *Brucella *sequence and annotation information for BGBrowser are stored in the database server instead of flat files. Both InterBru and BGBrowser share the same output page of detailed gene information (Figure 2C).

### Blast@BBP

The Blast module in BBP uses the latest web server version of BLAST obtained from NCBI [43]. It includes regular BLAST services (blastn, blastp, blastx, tblastn, tblastx), PSI/PHI BlAST, Mega BLAST, and BLAST 2 sequences. These services are implemented in the BBP application server and can be used to search nucleotide or protein BLAST libraries containing sequences from individual or combined *Brucella *genomes. The sequence libraries are updated periodically to reflect newly curated annotations and when new genomes are added.

### TextPresso-powered *Brucella *literature search

As a software program from the GMOD, Textpresso uses a modified GNU license and is free for academic purposes [24]. The TextPresso package is downloaded from the TextPresso website [24]. An automatic download program is first used to download and extract from PubMed all *Brucella*-related article information, including titles, authors, publish years, volumes, pages, journal names and abstracts. A BBP script is also developed to extract all *Brucella*-related full-text PDF files from PubMed. The PDF files are converted into plain text files using the open source XPDF [44]. The converted full text together with abstracts and titles are tokenized into sentences and then to XML-tagged words or phrases representing different ontology categories according to a pre-defined ontology format. All the processed information, including fully annotated abstracts, titles, full texts, citation information, authors, years, keywords and categories, is indexed for efficient query. A web query interface is installed and modified for users to search against the indices and check detailed matching records.

### *Brucella *Limix

To develop the literature mining and curation system, the PubSearch version 0.81 is first adopted and extended from GMOD [22]. PubSearch is originally designed for Arabidopsis in the TAIR project [17]. We replace the TAIR data from the software download with new *Brucella *genomic data from NCBI, Swiss-Prot and other repositories. Currently Limix stores information for 6033 *Brucella*-related articles downloaded from PubMed, including those without abstract content. The 20346 GO ontology terms downloaded from the GO database [45] allow users to associate *Brucella *gene names with specific GO terms. Limix also includes batch mode loading of data from other databases (e.g., PubMed and GO databases), and data indexing. We have also modified many PubSearch features to make it fit in with bacterial genome annotation. The PubSearch-powered page programmed in Java is used as the primary Limix web page specifically for manual curation and management. Since TextPresso uses Perl CGI instead of Java, we use a HTML frame inside the primary page to hold the TextPresso-powered computational text mining program (Figure 4). The text mining HTML frame also contains literature keywords search and automatic *Brucella *literature update programs. A JavaScript program is developed to copy highlighted sentences from the text mining frame to an editable text field in the primary curation page.

### MeSH Browser

The *Brucella *literature MeSH browser is developed by utilizing the hierarchy tree structure of MeSH terms downloaded from PubMed and stored in the BBP Oracle database. MeSH Browser allows users to search associated articles to a specific MeSH term in the MeSH tree by clicking and expanding the MeSH nodes. The nodes in the MeSH tree can be dynamically expanded with no waiting for pages to reload by using the Asynchronous JavaScript and XML (Ajax) technique [46].

### Publication email alert service and automatic updates

The BBP publication email alert service is initiated by a subscribed user to specify the notification frequency (daily, weekly, bimonthly or monthly) and the keywords to be searched against the PubMed database. A daily Linux cron job checks the subscription database, searches for updates in PubMed, and sends the updated paper notification to the users through email. The automatic literature update program allows all *Brucella*-related publications from the current and previous months automatically updated in the BBP website. It is implemented by dynamically querying PubMed for updated publications during a certain month when the page is opened. This program is also integrated into the *Brucella *Limix for data curators to obtain the newest publications not stored in local publication database.

### ContactsDB and forum

The ContactsDB database stores contact information of individual *Brucella *researchers in an Oracle database. A PHP program is developed for the users to query, submit, and update contact information in the BBP ContactsDB web page. The discussion forum program is also implemented with PHP and Oracle database.

## Abbreviations

Ajax Asynchronous JavaScript and XML

BBP *Brucella *Bioinformatics Portal

CDD The Conserved Domain Database

CMR TIGR Comprehensive Microbial Resource

COG The Clusters of Orthologous Groups

EC Enzyme Commission

GMOD Generic Software Components for Model Organism Databases

GO Gene Ontology

Limix Literature Mining and Curation System

MeSH Medical Subject Headings

MW Molecular weight

NCBI The National Center for Biotechnology Information

NIAID The National Institute of Allergy and Infectious Diseases

NIH National Institutes of Health

NLP Natural Language Processing

SOD Superoxide Dismutase

PI Isoelectric points

TIGR The Institute for Genomic Research

## Authors' contributions

ZX: Current webmaster, software programmer, and database administrator.

WZ: Previous webmaster, software programmer, and database administrator.

YH: Project initiator, designer, software programmer, and manager. As an active *Brucella *researcher, YH also curated all *Brucella *genetic interactions and mutations available in BBP using the *Brucella *Limix system.

## Supplementary Material

Additional File 1 BBP website screenshot. The image provided is the screenshot of the Brucella Bioinformatics Portal (BBP) website home page. The BBP URL is: . Click here for file

## Figures and Tables

**Table 1:** Public databases and software programs linked or used in BBP. Unique database identifiers (e.g., RefSeq ID) are usually stored for linking to public database web pages. *Brucella *literature abstracts and full text PDF files are also extracted from PubMed. Software programs are integrated into BBP in different ways.

Resources	**Databases and analysis programs**	**Comments**
Databases		
NCBI	PubMed	Biomedical publications
	MeSH	Medical Subject Headings
	RefSeq	Reference sequences
	Genome	Genome summary
	Gene	Gene information
	Protein	Protein information
	Nucleotide	Nucleotide information
	CDD	conserved domains
	COGs	clusters of orthologous groups
	Taxonomy	*Brucella *taxonomy information
	3D structure DB	3D structures (typically of related proteins)
	Feature tables	Protein coding genes, functional and structural RNAs,
EBI & SIB	Swissprot	Annotated protein data
	TrEMBL	protein data
	InterPro	protein families, domains and functions
	PROSITE	protein families and domains
TIGR	CMR	codon usage, EC numbers, condensed genome display, role category graph, GO terms, gene attribute, Role category gene list, RNAs, terminators
	TIGRfam	TIGRfam assignments
Others	PFam	protein domains and families
	ProDom	protein domain families
Software programs integrated		
NCBI	BLAST	Blastn, blastp, blastx, tblastn, tblastx, PSI/PHI Blast, Mega Blast, Blast 2 sequences
GMOD	GBrowse	genome browse and analysis
	TextPresso	NLP text mining
	PubSearch	literature curation management
Other	BioPerl	Programming tools

**Table 2:** Clustering of 75 attenuated *Brucella *genes found from literature search using the COG classification method.

**COG clusters**	**Mutated *Brucella *genes**
C: Energy production and conversion	4: *cydB*, *eryB*, *narG*, *pyc*
D: Cell cycle control, mitosis and meiosis	1: *virB5*
E: Amino acid transport and metabolism	15: *aroC*, *aspC*, *gcvP*, *gcvT*, *glnA*, *glyA*, *hisC*, *hisD*, *hisF*, *leuA*, *leuC*, *lysA*, *pepN*, *pheA*, *serB*
F: Nucleotide transport and metabolism	10: *dut*, *purD*, *purE*, *purF*, *purH*, *purl*, *purM*, *purN*, *pyrB*, *pyrD*
G: Carbohydrate transport and metabolism	7: *eryC*, *gnd*, *ilvD*, *pgi*, *pgm*, *ugpA*, *zwf*
H: Coenzyme transport and metabolism	3: *cobB*, *hemH*, *ilvC*
I: Lipid transport and metabolism	2: *bacA*, *uppS*
J: Translation	3: *pth*, *rplS*, *rpsA*
K: Transcription	3: *miaA*, *oxyR*, *rpoA*
L: Replication, recombination and repair	2: *recA*, *xseA*
M: Cell wall/membrane biogenesis	6: *gmd*, *omp10*, *omp19*, *omp25*, *virB1*, *wboA*
O: Posttranslational modification, protein turnover, chaperones	5: *cydD*, *degP*, *dnaK*, *lon*, *tig*
P: Inorganic ion transport and metabolism	2: *sodC*, *znuA*
Q: Secondary metabolites biosynthesis, transport and catabolism	1: *pncA*
R: General function prediction only	2: *gltD*, *hfq*
T: Signal transduction mechanisms	4: *bvrR*, *bvrS*, *ntrC*, *ntrY*
U: Intracellular trafficking and secretion	9: *fliF*, *virB2*, *virB10*, *virB11*, *virB3*, *virB4*, *virB6*, *virB8*, *virB9*
-: Not in COGs	6: *cowB*, *glnD*, *gluP*, *gpt*, *norM*, *wbkB*

**Table 3:** TextPresso-predicted and manually curated *Brucella *genetic interactions. One match means one highlighted sentence containing at least 2 genes and at least one word under "association" or "regulation" category. Each match represents for one predicted genetic interaction. The results are shown by manually verified vs. TextPresso-predicted interactions. The number of verified vs. predicted interactions varies depending on the numbers (#) of matches and papers to use as the cutoffs and whether or not to use full text contents besides paper abstracts.

**Abstract**							**Full text (containing abstract)**							
# of papers	# of matches						# of papers	# of matches						
			
	1	2	3	4	5	10		1	2	3	4	5	10	20

1	17/38	6/10	1/1	1/1	1/1	0/0	1	58/1330	50/213	42/105	34/81	31/63	16/26	6/7
2	3/5	3/5	1/1	1/1	1/1	0/0	2	46/172	46/172	41/94	34/73	31/55	16/26	6/7
3	1/1	1/1	1/1	1/1	1/1	0/0	3	33/71	33/71	33/71	30/60	28/45	15/24	6/7
4	1/1	1/1	1/1	1/1	1/1	0/0	4	28/50	28/50	28/50	28/50	27/40	15/23	6/7
8	0/0	0/0	0/0	0/0	0/0	0/0	8	8/10	8/10	8/10	8/10	8/10	8/10	6/7
