# Supplementary figures and images for: BBP: Brucella genome annotation with literature mining and curation
Source: BMC Bioinformatics. 2006 Jul 16;7:347. doi: 10.1186/1471-2105-7-347 (PMC1539029; doi:10.1186/1471-2105-7-347)

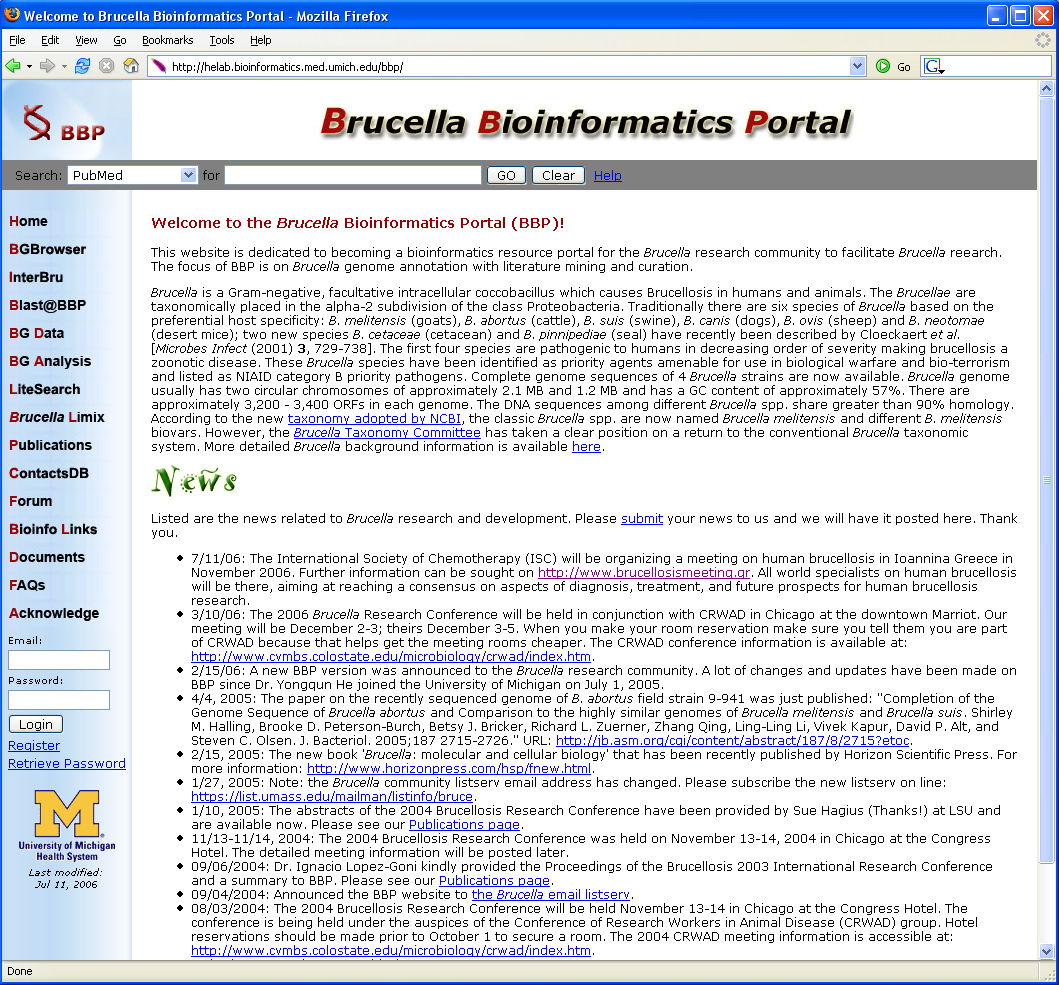

Supplement: Additional File 1 — BBP website screenshot. The image provided is the screenshot of the Brucella Bioinformatics Portal (BBP) website home page. The BBP URL is: . [file 1471-2105-7-347-S1.tiff]
